# Development and Evaluation of Novel Water-Based Drug-in-Adhesive Patches for the Transdermal Delivery of Ketoprofen

**DOI:** 10.3390/pharmaceutics13060789

**Published:** 2021-05-25

**Authors:** Kwanputtha Arunprasert, Chaiyakarn Pornpitchanarong, Theerasak Rojanarata, Tanasait Ngawhirunpat, Praneet Opanasopit, Porawan Aumklad, Prasopchai Patrojanasophon

**Affiliations:** 1 Pharmaceutical Development of Green Innovations Group (PDGIG), Faculty of Pharmacy, Silpakorn University, Nakhon Pathom 73000, Thailand; arunprasert_k@su.ac.th (K.A.); pornpitchanaron_c@su.ac.th (C.P.); rojanarata_t@su.ac.th (T.R.); ngawhirunpat_t@su.ac.th (T.N.); opanasopit_p@su.ac.th (P.O.); 2 OLIC (Thailand) Limited, Bang Pa-in, Ayutthaya 13160, Thailand; porawan.a@olic-thailand.com

**Keywords:** ketoprofen, *N*-vinylpyrrolidone-co-acrylic acid, water-based adhesive, transdermal patches

## Abstract

The objective of this study was to develop novel water-based drug-in-adhesive pressure-sensitive adhesives (PSAs) patches for the transdermal delivery of ketoprofen, employing poly(*N*-vinylpyrrolidone-co-acrylic acid) copolymer (PVPAA) and poly(methyl vinyl ether-alt-maleic anhydride) (PMVEMA) as the main components. The polymers were crosslinked with tartaric acid and dihydroxyaluminium aminoacetate using various polymer ratios. Ketoprofen was incorporated into the PVPAA/PMVEMA PSAs during the patch preparation. The physicochemical properties, adhesive properties, drug content, release profile, and skin permeation of the patches were examined. Moreover, the in vivo skin irritation and skin adhesion performance in human volunteers were evaluated. The patches prepared at a weight ratio of PVPAA/PMVEMA of 1:1 presented the highest tacking strength, with desirable peeling characteristics. The ketoprofen-loaded PVPAA/PMVEMA patches exhibited superior adhesive properties, compared to the commercial patches, because the former showed an appropriate crosslinking and hydrating status with the aid of a metal coordination complex. Besides, the permeated flux of ketoprofen through the porcine skin of the ketoprofen-loaded PVPAA/PMVEMA patches (4.77 ± 1.00 µg/cm^2^/h) was comparable to that of the commercial patch (4.33 ± 0.80 µg/cm^2^/h). In human studies, the PVPAA/PMVEMA patches exhibited a better skin adhesion performance, compared with the commercial patches, without skin irritation. In addition, the patches were stable for 6 months. Therefore, these novel water-based PSAs may be a potential adhesive for preparing drug-in-adhesive patches.

## 1. Introduction

Currently, transdermal drug delivery systems (TDDS) are an attractive alternative drug delivery pathway for oral administration and hypodermic injection, as they are painless, convenient, and may increase the bioavailability of the drugs [1]. Moreover, TDDS may provide the controlled delivery of a drug through the stratum corneum for local or systemic approaches [2]. TDDS provides several advantages, such as the avoidance of drug destruction in the digestive tract and hepatic first-pass effect, a reduction of gastrointestinal side effects, a single application for multi-day therapy, the possibility of rapid cessation due to an uncomplicated removal, and the possibility of providing an accurate dose of drugs [3]. Drug-in-adhesive patches take advantage of close contact by the attachment of the patches and the stratum corneum, the barrier to drug transportation through the skin, to deliver the drugs into the skin or the blood circulation [4]. Pressure-sensitive adhesives (PSAs) are one of the most significant components in a transdermal system. PSAs are used for skin adhesion and as a matrix for drugs and other excipients. PSAs can impact the properties of transdermal patches, such as drug permeation, adhesive properties, and physicochemical properties. PSAs should be safe for the skin, adhere quickly and strongly to the skin, and be detachable without pain and leave no residue on the skin [5].

Conventional PSAs can be divided into three types, including polyisobutylenes (PIBs), silicones, and acrylics [6]. The choice of PSA is based on the physicochemical properties of the drug and the patch types [7]. Nevertheless, conventional PSAs have some problems, such as the thermal instability of polyisobutylene adhesives, the expensive cost of silicones, the low water permeation of hydrophobic PSAs, inadequate adherence to the skin surface, and environmental pollution from organic solvent residues. Therefore, innovative PSAs should be developed to increase the quality and efficiency of transdermal patches [8]. Water-based or hydrogel PSAs are well-defined as water-absorbed polymeric networks with a high water content [9]. The polymeric crosslink is created by the integration of hydrophilic polymers. It is naturally compatible with many drugs and can moisten the skin, thus leading to an effective transdermal patch, without the use of a permeation enhancer [7]. Only a few investigations are available on the development of hydrogel PSAs. Bai et al. proposed hydrophilic PSAs containing ferulic acid. VISCOMATE™ NP700 (neutralized sodium polyacrylic acid), tartaric acid (TA), glycerine, and dihydroxy aluminium aminoacetate (DAAA) were used in different proportions in the formulation of the hydrophilic PSAs [10]. Furthermore, poly(sodium methacrylate, methylmethacrylate) hydrophilic PSAs were developed for lidocaine hydrochloride. Polyethylene glycol, glycerin, sorbitol, and sodium chloride were added as excipients in the PSAs for transdermal patches [11].

*N*-vinylpyrrolidone (NVP) and acrylic acid (AA) are water-soluble monomers, which are commonly used to produce a polymer with a high biocompatibility and low toxicity [12]. Chain polymerization techniques are used to synthesize poly (*N*-vinylpyrrolidone-co-acrylic acid) (PVPAA) polymeric nanoparticles (NPs) from NVP and AA to deliver cisplatin for the treatment of oral cancer [12]. Poly(methyl vinyl ether-alt-maleic anhydride) (PMVEMA) is a water-soluble, biocompatible, and nontoxic polymer. It has been applied as a film-forming agent, composition in toothpaste, and moisture-stimulated bio-adhesive material. The hydroxyl groups of PMVEMA can also form interpolymer complexation with the proton-accepting groups, such as ether or carbonyl groups. This complexation may critically impact the adhesive properties and drug release of the finished products [13].

Ketoprofen is an outstanding candidate for transdermal delivery among the various non-steroidal anti-inflammatory drugs (NSAIDs), as it has an optimum solubility and partition coefficient, compared to other NSAIDs [14,15]. Ketoprofen is given by oral dosage forms at 300 mg/day [16]. In addition, the oral administration of ketoprofen might induce side effects involving the gastrointestinal systems, such as digestive bleeding, intestinal burning, and ulcerations [17,18]. The use of NSAIDs in the form of analgesic patches is becoming widespread. The drug is delivered across the skin to the targeted site to avoid the gastrointestinal side effects [19].

Herein, PVPAA was synthesized from NVP and AA using surfactant-free emulsion polymerization. The successful synthesis was confirmed using Fourier-transform infrared spectroscopy (FT-IR) and proton nuclear magnetic resonance spectroscopy (^1^H-NMR) spectra. The cytotoxicity of the synthesized polymer was investigated by an MTT assay on normal human skin fibroblast cells (HSF). The water-based PSAs were produced by crosslinking reaction through the metal-coordination complex between PVPAA and PMVEMA at different weight ratios, using TA and DAAA as crosslinking agents. The adhesive properties of the PVPAA/PMVEMA PSA patches were evaluated by measuring the tacking and peeling strength. Ketoprofen-loaded PVPAA/PMVEMA patches were prepared. The physical appearance and color, drug content, adhesive properties, and drug permeation of the transdermal patch were studied and compared with the commercial product. Besides, the in vivo skin irritation and skin adhesion performance were evaluated in human volunteers.

## 2. Materials and Methods

### 2.1. Materials

AA (AA 99%, containing 250 ppm monomethyl ether hydroquinone (MEHQ) as inhibitor), NVP, methylthiazolyldiphenyl tetrazolium bromide (MTT), 2,2′-azobis(2-methylpropionamidine) dihydrochloride (V50), and ketoprofen were acquired from Sigma Chemicals (St. Louis, MO, USA). PMVEMA was bought from Ashland (Wilmington, DE, USA). Ketoprofen transdermal patches were a gift from Hisamitsu^®^ Pharmaceutical Co., Inc. (Chiyoda-ku, Tokyo, Japan). DAAA was acquired from Kyowa Chemical Industry Co., Ltd (Chou-ku, Tokyo, Japan). TA was purchased from Tartaros Gonzalo Castello SL (Novelda, Alicante, Spain). Glycerin was purchased from Emery Oleochemicals (Telok Panglima Garang, Selangor, Malaysia). Non-woven fabric SBP 100 gsm and silicone-coated polyester were a gift from Polyplex (Pluak Daeng, Rayong, Thailand). Deuterium oxide (D_2_O) was acquired from Cambridge Isotope Laboratories (Tewksbury, MA, USA). Dulbecco’s modified Eagle’s medium (DMEM), fetal bovine serum (FBS), trypsin–ethylenediaminetetraacetic acid (EDTA), and penicillin-streptomycin were purchased from Gibco BRL (Rockville, MD, USA). Human skin fibroblast (HSF) cells were bought from the American-Type Culture Collection (ATCC) (Rockville, MD, U.S.A.). Abdominal porcine skin was obtained from intrapartum stillborn animals, from a farm in Nakhon Pathom, Thailand. Other chemicals and reagents were used as purchased, without further purification.

### 2.2. Synthesis of PVPAA

PVPAA was synthesized using a surfactant-free emulsion polymerization reaction between NVP and AA. The synthesis was modified from a previously reported procedure [12]. Briefly, 0.05-0.2%wt V50 (the initiator) was dissolved in 50 mL of deionized water heated at around 70 °C. The mixture was purged with nitrogen gas to create an inert environment. AA and NVP (3:1 by mole) were added to a round-bottomed flask and mixed. The reaction was conducted for 18 h. Thereafter, the unreacted compounds were eliminated by dialysis (a molecular weight cut-off of 3500 Da), with a water replacement every 6 h for 3 days. The synthesized polymer was collected after lyophilization.

### 2.3. Characterization of PVPAA

#### 2.3.1. H-NMR

Structural elucidations of the PVPAA were carried out by ^1^H-NMR. The analysis was performed at 298 K under a 300 MHz NMR spectrometer, AVANCE III HD (Bruker, Billerica, MA, USA). The spectra were recorded and reported as the chemical shifts (δ) in part per million (ppm), using D_2_O as a solvent reference (δ = 4.80 ppm).

#### 2.3.2. ATR-FTIR

The chemical structures of PVPAA were characterized using ATR-FTIR (Nicolet iS5, Thermo Fisher Scientific, MA, USA). The investigation was performed at 16 running scans and 4 cm^−1^ resolutions. The spectra were collected from 400–4000 cm^−1^.

#### 2.3.3. Cytotoxicity Evaluation of PVPAA

The cytotoxicity of the synthesized PVPAA was verified using an MTT assay. Human skin fibroblast (HSF) cells in Dulbecco’s modified Eagle’s medium (DMEM) were allocated into 96-well plates (10,000 cells/well). The cells were cultivated under 37 ˚C, with 95% air and 5% CO_2_. Then, the cells in DMEM without serum were incubated with PVPAA polymers at concentrations ranging from 1 to 3,000 µg/mL for 24 h. Afterward, PBS pH 7.4 was used to rinse the cells, before serum-supplemented DMEM medium (100 µL) and MTT solution (1 mg/mL, 25 µL) were added. After 3 h, the precipitate of formazan formed after the incubation was dissolved by adding DMSO (100 µL), and the absorbance was detected at 550 nm (VICTOR Nivo^TM^, Perkin Elmer, MA, USA). The calculation of the % relative cell viability was conducted following Equation (1):

(1) $$\mathrm{Relative}\;\mathrm{cell}\;\mathrm{viability}\;\left(\%\right)=\frac{{\mathrm{Abs}}_{550,\mathrm{sample}}-{\mathrm{Abs}}_{550,\mathrm{blank}}}{{\mathrm{Abs}}_{550,\mathrm{control}}-{\mathrm{Abs}}_{550,\mathrm{blank}}}\times 100$$

### 2.4. Preparation of Water-based PVPAA/PMVEMA PSAs

The PVPAA/PMVEMA PSAs were prepared from PVPAA and PMVEMA (at different weight ratios of the polymers, with the final concentration of the polymer mixture of 7%wt), using 0.2%wt DAAA and 0.1%wt TA as a crosslinking agent and a regulator of the crosslinking reaction, respectively. PVPAA was dissolved in water, while PMVEMA was firstly mixed with DAAA, TA, and 30%wt glycerin. Afterward, the PVPAA solution was added to the PMVEMA mixture at various weight ratios of PMVEMA to PVPAA (1:0, 1:1, and 2:1) and mixed thoroughly using a magnetic stirrer. The mixture was then cast on the backing cloth of non-woven fabric SBP 100 gsm with a size of 4 × 4 cm^2^. The casted PVPAA/ PMVEMA patches were then cured at room temperature for 24 h. After curing, the patches were covered with a release liner made of silicone-coated polyester. The drug-in-adhesive PVPAA/PMVEMA transdermal patches were prepared by dissolving 0.3%wt ketoprofen in ethanol, before adding it to the polymer mixture. The physical appearance of the prepared hydrogel patches was investigated as the color of the hydrogel.

### 2.5. Adhesive Evaluation

#### 2.5.1. Tacking Strength

A texture analyzer (TA.XTPlus, Stable Micro Systems, Godalming, UK) was used to examine the tacking strength of the transdermal patches. The release liners of the prepared patches were removed immediately before the test. In this test, the cylindrical Perspex probe (P 0.5/R) of the texture analyzer, coupled with a 5-kg load cell, was lowered down at a rate of 1 mm/sec to press on the patches with a force of 5 N. Then, the probe was pulled up at a rate of 0.5 mm/sec. The maximum force required to pull the probe away from the adhesive was recorded as the tacking strength (N).

#### 2.5.2. Peeling Strength

Measurement of peeling strength was performed to ensure that the adhesives did not damage the skin, and there were no residues left on the skin after peeling. The test was conducted following a 180° peel strength test method, using a texture analyzer (TA.XTPlus, Stable Micro Systems, Godalming, UK) with a 5-kg load cell and a tensile grips holder. The release liner of the prepared patch was removed immediately before the test. One end of the tested patch was applied to the tested platform, and the other end was attached to the grip holder. After that, the patch was peeled from the tested platform in the horizontal direction (180° angle) at a pulling rate of 2 mm/min. The maximum force required to peel the patch from the platform was recorded.

### 2.6. Metal-Coordination Complex Formation of the Hydrogel PSAs

The formation of a three-dimensional network of a hydrogel is potentially associated with the creation of metal-coordination bonds between aluminum ions and carbonyl, carboxyl, and ether group of the copolymers [20], which was confirmed by ATR-FTIR (Nicolet iS5, Thermo Fisher Scientific, MA, USA). Furthermore, to prove the crosslinking mechanism of the metal-coordination complex, strong acid (1 N HCl) and strong base (1 N NaOH) solutions were separately added to the PVPAA/PMVEMA [21]. The appearance of the samples was observed after immersing the polymer mixture in each solution.

### 2.7. Content Determination

Accurately weighed samples (with a size of 2 × 2 cm^2^) were soaked in methanol at room temperature for 24 h. An Agilent Infinity Series 1200 HPLC (Agilent Technologies, Santa Clara, CA, USA) was used to determine the amount of ketoprofen in the patches. A C18 column (column dimension 4.6 × 150 mm, with a particle size of 5 μm) was used as a stationary phase, with an injection volume of 20 μL. The mobile phase consisted of methanol and 0.1% *v/v* phosphoric acid (75:25), and the flow rate was set at 1 mL/min. The detection was performed at 254 nm using a UV detector [22]. The standard curve of ketoprofen was prepared at a concentration range of 0.5–100 μg/mL, and the *R*^2^ value was 0.999.

### 2.8. In Vitro Skin Permeation

#### 2.8.1. Skin Preparation

A stillborn pig was received from a slaughterhouse in Nakhon Pathom, Thailand, on the day of slaughter. The abdominal area of the skin samples was cut. After that, the adherent subcutaneous layer, the hair on the selected site, and other debris were gradually removed. The skin was kept in phosphate buffer saline (PBS, pH 7.4) and stored in a freezer until further use.

#### 2.8.2. In Vitro Skin Permeation Studies

The in vitro permeability of ketoprofen from the PVPAA/PMVEMA transdermal patches was investigated on stillborn porcine skin using Franz diffusion cells (1.77 cm^2^ in the area of the applied sample and 6 mL in the receptor chamber). The in vitro permeability was conducted at 37 ± 2 °C. A mixture of PBS (pH 7.4) and methanol (1:1) was added to the receptor compartments as the medium. Small magnetic bars were put in receptor chambers to stir the medium. The prepared skin pieces were applied over the receptor diffusion cell compartment, and the air bubbles in the medium were removed. The commercial patches and ketoprofen-loaded patches were cut to 1.77 cm^2^ and were adhered to the prepared skin. The solutions from the receptor fluid were collected (0.5 mL) at 0, 1, 2, 3, 4, 6, 8, and 24 h, and the same volume of fresh medium was added to the receptor chamber to maintain a steady volume. The amount of permeated ketoprofen at each time point was quantified by HPLC using the method mentioned in Section 2.7 (Content determination). The cumulative amount of ketoprofen permeated through the porcine skin was plotted against the time, and the steady-state flux was determined as the slope of the linear regression. The skin permeation of ketoprofen was analyzed using a mathematical model based on Fick’s law of diffusion following Equation (2):

(2) $$K_{p}=\frac{J}{C_{d}}$$

where *J* is the steady-state flux, *C_d_* is the donor concentration of the formulations, and *K_p_* is the permeability coefficient.

### 2.9. Stability Studies

Stability studies of the selected ketoprofen-loaded PVPAA/PMVEMA patches were conducted for 6 months, according to the condition specified by the ICH guidelines under an accelerated condition (40 ± 2 °C, 75 ± 5% RH) and long-term condition (30 ± 2 °C, 65 ± 5% RH) to confirm the stability of the patches in terms of physical appearance, adhesive properties, and drug content.

### 2.10. In Vivo Skin Irritation Test

A skin irritation study was conducted in 24 healthy human volunteers (aged between 25 and 35 years old), who agreed to participate in the study. This study was approved by an Investigational Review Board (Human Studies Ethics Committee, the Research, Innovation and Creativity Administration Office, Sanam Chandra Palace Campus, Silpakorn University; approval no REC 63.1113-141-6391; approval date: 26 January 2021). For at least 12 h before the measurements, the subjects did not use any moisturizer products. The ketoprofen-loaded PVPAA/PMVEMA patch and the commercial patch (size 2 × 2 cm) were applied randomly at contralateral locations of the same anatomical site (left arm and right arm). After applying these formulations for 24 h, the DermaLab^®^ series (SkinLab Combo, Cortex Technology, Hadsund, Denmark) was used to evaluate the percentage erythema index value of skin, and the normal skin vascularity equated to 100%, as shown in Equation (3):

% EI = 100 + ((*E_t_* − *E*)/*E*) × 100 (3)

where *E_t_* is the erythema value of treated skin, and *E* is the erythema value of untreated skin.

### 2.11. Safety Evaluation and In Vivo Skin Adhesion Performance

Twenty-three subjects were included in this study. The subjects did not use any moisturizer products 12 h before the test. The ketoprofen-loaded PVPAA/PMVEMA patch and the commercial patch (size 2 × 2 cm) were applied randomly at contralateral locations of the same anatomical site (left arm and right arm). The fall-off time of each patch was recorded for the evaluation of the adhesive performance. A high value of fall-off time indicates great adhesive properties of the transdermal patch.

### 2.12. Statistical Analysis

All experiments were performed in triplicate. The numerical data were reported as the mean ± standard deviation (SD). The analysis of variance and comparisons were analyzed at a 95% confidence interval with an F-test and independent *t*-test, respectively. A *p*-value < 0.05 indicates a significant difference.

## 3. Results and Discussion

### 3.1. Synthesis of PVPAA

**Figure 1:**
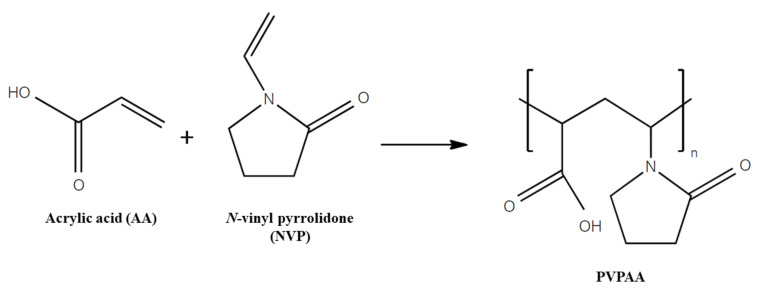
Synthetic pathway of PVPAA.

PVPAA copolymer was synthesized by a polymerization reaction between NVP and AA at a monomer molar ratio of 1:3. A previous study found that the % yield of this ratio was almost twice as great as that obtained at a monomer ratio of 1:1 [12]. The synthesis scheme is presented in Figure 1. The reaction was generated with the free radical initiator (V50). The percentage of an initiator used in the synthesis reaction was varied. The % yields of the PVPAA, prepared using different portions of V50 as an initiator, are listed in Table 1. The results revealed that once the percentage of V50 in the reaction was increased, the %yield of PVPAA decreased. For this reason, the PVPAA-01 provided the highest yield. This may be because the excess amount of initiator led to a rapid chain termination of the polymer during the polymer synthesis, providing short-chain polymers [23]. Consequently, the short polymer chains might be removed during the purification process to achieve a low percent yield. Therefore, PVPAA-01, which provided the highest yield, was then selected for further experiments.

**Figure 2:**
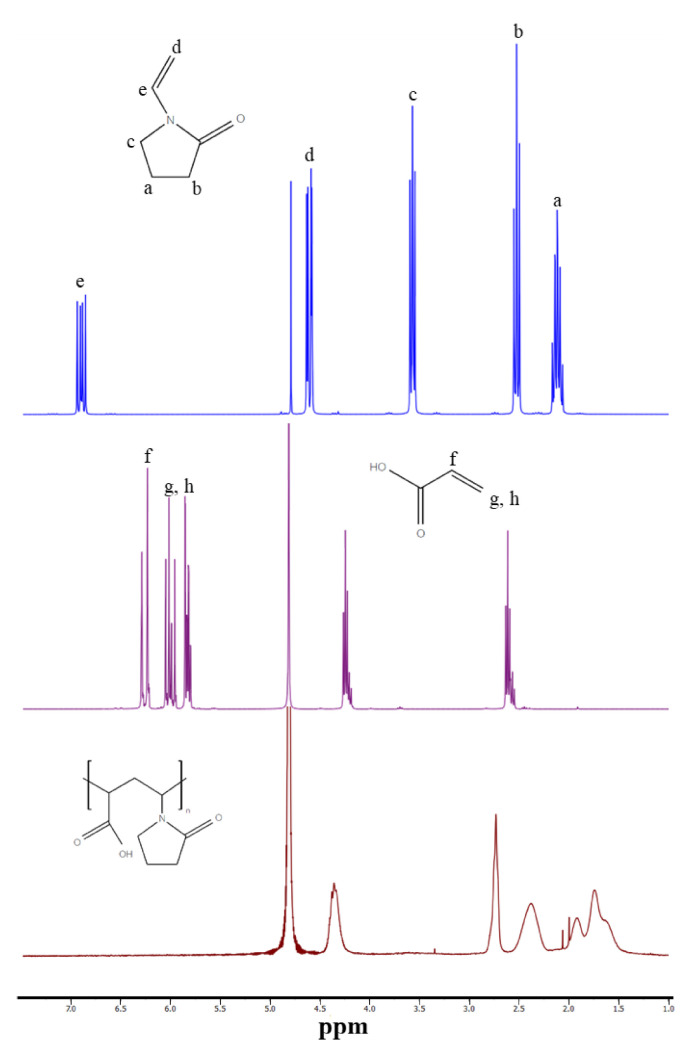
^1^H-NMR Spectra of NVP, AA, and the synthesized PVPAA.

**Figure 3:**
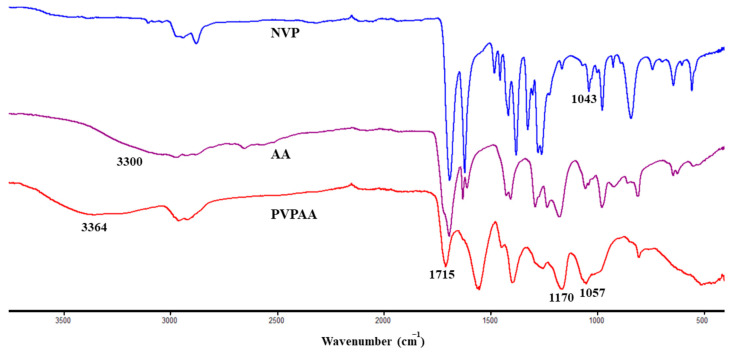
FT-IR Spectra of NVP, AA, and the synthesized PVPAA.

The synthesized PVPAA were characterized for the structural attributes using ^1^H-NMR and ATR-FTIR. From the ^1^H-NMR spectra (Figure 2), the protons at the a, b, and c positions of amide in the NVP structure were presented as multiplets at 2.1, 2.5, and 3.6 ppm, respectively. The protons at the d and e positions of the NVP structure were presented as multiplets at 4.6 and 6.9 ppm, respectively. In the structure of acrylic acid, the protons at the f, g, and h positions were exhibited as multiplets at 6.2, 6, and 5.8 ppm, respectively. The polymerization reaction was performed by the decomposition of the free-radical initiator at its decomposition temperature, creating free radical species. The radicals initiate polymer chain propagation via an electron transfer at the double bond of AA and NVP. During chain lengthening by monomer addition, the vinyl group of AA and NVP switched to a single bond connected to the joining monomer, resulting in a single bonded polymer chain of PVPAA. Therefore, the protons at the d, e, f, g, and h positions, which were attributed to the protons of the AA and NVP vinyl groups, disappeared in the spectrum of the PVPAA and were altered to a multiplet at 1.5–2.9 ppm. However, the proton at the a, b, and c positions remained. Figure 3 shows the ATR-FTIR spectra of the synthesized PVPAA. A broad peak of the O–H stretching of the hydroxyl groups of AA was presented at 3364 cm^−1^. The strong carboxylic carbonyl and C–O stretching were represented by the peaks at 1715 and 1170 cm^−1^, respectively. The C–N stretching at 1057 cm^−1^ corresponds to the pyrrolidone ring of the NVP. The spectra of both spectrometric investigations confirmed the expected structure of the synthesized PVPAA.

**Figure 4:**
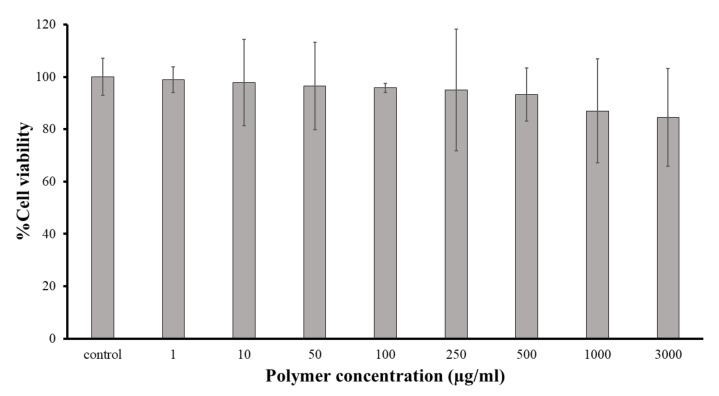
Percentage cell viability of the synthesized PVPAA on HSF cells.

To apply the synthesized polymer as PSAs, the polymer should be safe and biocompatible. The in vitro cytotoxicity was assessed using an MTT test. The PVPAA polymer solutions, with concentrations ranging from 1 to 3000 μg/mL, were prepared in the culture medium and were incubated with HSF cells for 24 h. The percentage of cell viability of the HSF cells after being incubated with the synthesized polymers compared with the untreated control cells is displayed in Figure 4. The results showed that all the tested polymers were nontoxic at the tested conditions, and the cell viabilities were higher than 80%, compared with the untreated control cells. Essentially, the safety of the monomers used has been previously reported, and they are widely used in drug delivery systems [24,25,26].

### 3.2. Preparation and Metal-Coordination Complex Formation of the PVPAA/PMVEMA PSAs

To prepare water-based PVPAA/PMVEMA PSAs, the synthesized PVPAA was crosslinked to PMVEMA, using TA as a regulator of crosslinking and DAAA as a crosslinking agent. The physical appearance of PVPAA/PMVEMA PSAs, prepared using various weight ratios of PVPAA/PMVEMA, was found to be a colorless sticky hydrogel. A previous study reported that a strong hydrogen bond is formed by the interaction of NVP and AA. These monomers can also interact via ion-dipole and ion-ion bonds, especially in a partially neutral condition [27]. Furthermore, an acrylic copolymer containing tertiary amine groups can enhance their cohesive strength [28]. Apart from the PVPAA copolymer, the crosslinking of PVPAA and PMVEMA can also increase the adhesive properties of PVPAA/PMVEMA PSAs, which was investigated in the following studies.

**Figure 5:**
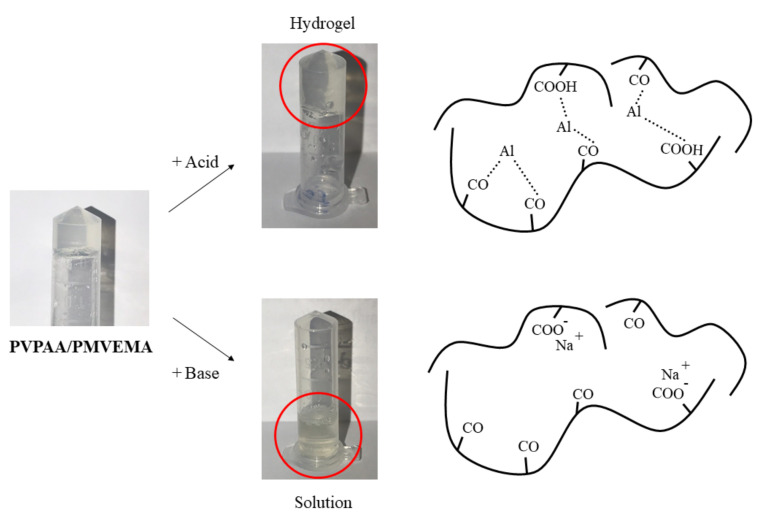
The appearance of the PVPAA/ PMVEMA in acidic and basic conditions.

**Figure 6:**
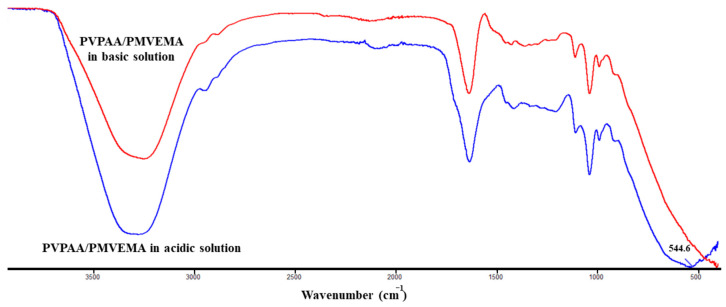
FT-IR Spectra of the PVPAA/ PMVEMA in acidic and basic conditions.

The crosslinking phenomenon between DAAA as the crosslinking agent and PVPAA/ PMVEMA is suggested to be due to the metal coordination complex. The hydronium ions that occurred after the dissociation of TA in water may take over the hydroxides from the crosslinker, influencing the availability of aluminum to form a metal coordination complex with PVPAA/PMVEMA. The representation of the coordination complexes is shown in Figure 5. The PVPAA/PMVEMA patches were separately submerged in a strong acid and strong base. The result showed that the hydrogel patches were able to remain in the initial state after being soaked in strong acid. However, the hydrogel patches were altered to the solution state in a strong base. This may be due to the reversion of hydroxides provided by the strong base to the crosslinker molecules, causing a loss of the metal coordination complex. A coordination complex is the product of a Lewis acid-base reaction, in which neutral molecules or anions (ligands) are bound to a central metal atom [29]. In the existence of a certain amount of water, TA protons promoted the dissociation of DAAA to be crosslinked with PVPAA/PMVEMA [10]. The hydrogel has more carbonyl, carboxyl, and ether groups in the molecular structure, which can form coordination bonds with aluminum ions [30]. Moreover, Figure 6 shows the ATR-FTIR spectra of the PVPAA/ PMVEMA in acid and base conditions. A broad peak of Al–O stretching of the metal-coordination complex of PVPAA/PMVEMA in the acid condition was exhibited at 544.6 cm^−1^ [20], indicating that the coordination complexes of the hydrogel patches were maintained in the acid condition. However, the Al–O stretching peaks in the spectrum of the PVPAA/PMVEMA disappeared after the patches were submerged in a strong base, and the three-dimension networks of the hydrogels were deformed. The appearance and the spectra of the hydrogel patches in both conditions verified the presence of metal-coordination bonds between aluminum ions and PVPAA/PMVEMA. Among the multiple weak non-covalent interactions, the metal-coordination complex is the most attractive, as the coordinative bond is relatively strong [31]. For this reason, the PVPAA/PMVEMA patch could provide strong adhesive properties for transdermal patches [32].

### 3.3. Adhesive Evaluation

The adhesive properties of the PVPAA/PMVEMA PSAs were evaluated by measuring the tacking and peeling strength, and the findings are presented in Table 2. Among the composite PVPAA/PMVEMA PSAs, the patches prepared at a weight ratio of PVPAA:PMVEMA of 1:1 provided the highest tacking strength, followed by those prepared at a weight ratio of 1:2 and 0:1, respectively. However, there was no significant difference in the peeling strength of all the patches. Therefore, the ratio of PVPAA: PMVEMA of 1:1 (F2) was selected for the preparation of the ketoprofen-loaded drug-in-adhesive patches. Interestingly, it was found that the ketoprofen-loaded PVPAA/PMVEMA patches exhibited comparable adhesive properties to the blank PVPAA/PMVEMA patches (F2). Moreover, ketoprofen-loaded patches were found to be significantly stickier than the commercial patches (Hisamitsu^®^). The hydroxyl groups of PMVEMA and the carbonyl groups of the PVPAA can create an interpolymer complex via hydrogen bonding. The interpolymer complexation was also found to increase the adhesive properties of the transdermal patch [13]. However, the highest adhesion strength was observed at the optimal ratio of the polymers. Discomfort to the skin due to the peeling of an adhesive is related to the force used to pull the transdermal patch from the skin. From the literature, the peeling strength should be less than 3.7 N, which indicates a painless removal of the patches [33]. While a significant difference in the tacking strength of F1, F2, and F3 was detected, no significant difference was observed in the peeling strengths of all formulations. Ideally, a stronger adhesion force should also present a stronger peeling force. Nevertheless, a study reported that as the peeling angle increases, the peeling force decreases [29]. In this work, a 180° angle was used in the peeling test, which is the recommended angle to pull off the patch in parallel to the skin to avoid pain. The adhesive force created at the interfacial layer between the PSAs and the skin surface could be stronger than the cohesion force of the patch, where residues of the materials may be left on the surface upon removal [30]. Therefore, the force obtained from the peeling test could have resulted from both the cohesion and adhesion force, rather than the latter alone, along with the higher examined angle, leading to the unrelated values from the tacking test. Above all, the patches could be removed from the skin without any skin damage due to the low peeling strength.

### 3.4. Drug Content Assay

The drug content of the drug-loaded patches and the commercial patches is presented in Table 3. Ketoprofen extracted from the drug-loaded patches, calculated as a percentage of the initial drug amount added to the patches, was found to be 98.98 ± 0.58% of the total amounts added, while the drug content of the commercial patches was found to be 98.78 ± 0.97%. These results indicated that the patch preparation process did not result in a significant loss of ketoprofen and showed a desirable drug recovery.

### 3.5. In Vitro Skin Permeation

**Figure 7:**
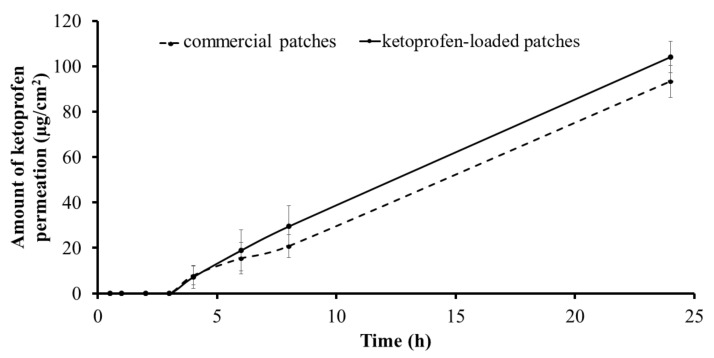
The skin permeation profiles and the skin permeation parameters of the drug-loaded patches and the commercial patches.

The skin permeation profiles and the skin permeation parameters of the ketoprofen-loaded PVPAA/PMVEMA patches and the commercial patches are displayed in Figure 7 and Table 3. The results revealed that there was no significant difference between the flux, lag time (3 h), and the permeability coefficient of ketoprofen from the drug-loaded patches (4.77 ± 1.00 µg/cm^2^/h) and those from the commercial patches (4.33 ± 0.80 µg/cm^2^/h). This finding revealed that ketoprofen-loaded patches could allow ketoprofen to penetrate the skin in a similar way to the commercial patches. Ketoprofen is a small molecule hydrophobic drug, with a logP value of approximately 3.1, which allows the drug to pass through the stratum corneum [34,35]. For the drug to pass through the model skin, it had to be released from the polymer matrix, which limited the permeation at the initial time points. Once the drug was released and passed through the skin, it was observed that the ketoprofen from the developed PSAs gradually passed through the skin barrier with a rate and extent comparable to that of the commercialized product, owing to its physicochemical properties. However, both formulations acted as drug reservoirs on the skin, with a certain force of adhesion to prolong the delivery of the drug. Despite containing the developed PVPAA/PMVEMA mixture in the patch, the component did not significantly affect the drug permeation profile, compared with the market product.

### 3.6. Stability Studies

The stability results are shown in Table 4. The results indicated that there was no significant difference in the physical appearance, tacking strength, peeling strength, and drug content of the ketoprofen-loaded PVPAA/PMVEMA patches determined at the beginning and at 3 and 6 months of the accelerated and long-term stability studies. Therefore, the ketoprofen-loaded PVPAA/PMVEMA patches were stable in terms of physical appearance, adhesive properties, and drug content for at least 6 months.

### 3.7. Safety Evaluation and In Vivo Skin Irritation Test

**Figure 8:**
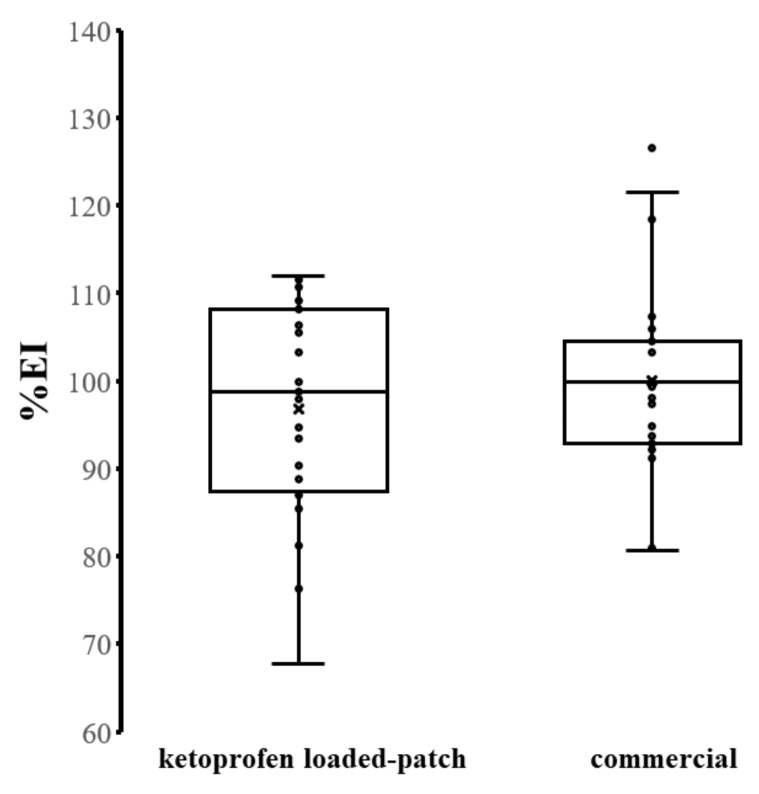
Percentage of the erythema index (%EI) of skin after a 24-h application of the drug-loaded patches and the commercial patches.

To evaluate the safety of the developed PVPAA/PMVEMA PSAs, the cytotoxicity of the synthesized polymer (PVPAA) was assessed, and the polymer was not toxic to the cells. The uses of other components of the PSAs in pharmaceutical and biomedical applications were previously reported. PMVEMA has been used in various oral formulations, while DAAA and TA were employed as crosslinkers in different medical preparations [13,36]. Cytotoxicity evaluation of the prepared PSAs may not be suitable due to the viscoelastic properties of the materials, which may affect the viscosity of the cell culture medium and the growth condition of the cells. To further elucidate the safety profile of the materials, an in vivo skin irritation test was also performed. After 24 h applications of the ketoprofen-loaded PVPAA/PMVEMA patch and the commercial patch, all subjects were monitored for skin changes in the erythema value. One subject was found to have itching symptoms when the commercial patch was applied. Thus, this participant was excluded. Figure 8 presented the percentage of the erythema index (%EI) of skin after a 24-h application of the ketoprofen-loaded PVPAA/PMVEMA patches and the commercial patches. Skin erythema is commonly characterized by a redness of the skin or mucous membranes, resulting from the hyperemia of superficial capillaries [37]. There was no significant difference in the %EI of the skin between the ketoprofen-loaded PVPAA/PMVEMA patches and commercial patches. Hydrogels have been reported to reduce skin irritation by absorbing moisture from the skin’s surface [38]. Consequently, the ketoprofen-loaded PVPAA/PMVEMA patch had a low toxicity to human skin and was possibly a material that can be used as a drug-delivering material for transdermal drug delivery systems.

### 3.8. In Vivo Skin Adhesion Performance

**Figure 9:**
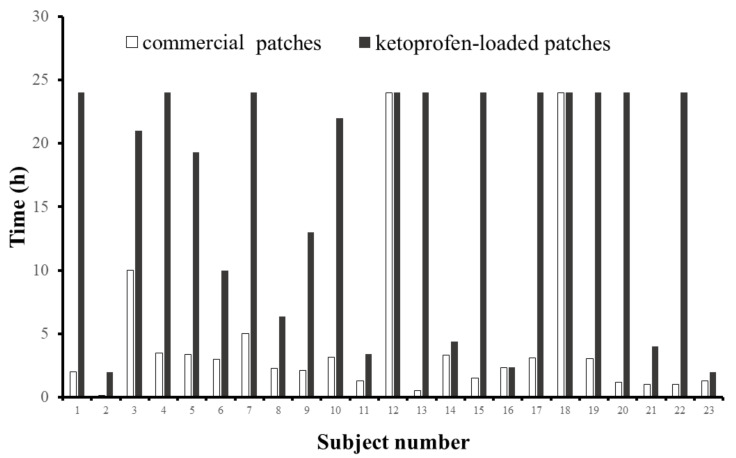
Fall-off time of the drug-loaded patches, compared with the commercial patches, in twenty-three subjects.

Twenty-three subjects completed the study. Figure 9 displays the fall-off time of the ketoprofen-loaded PVPAA/PMVEMA patches, compared with the commercial patches. A high value of fall-off time indicates great adhesive properties of the transdermal patch. Overall, the PVPAA/PMVEMA patches exhibited a better skin adhesion performance, compared with the commercial patches, in almost all subjects. The patches were able to adhere to the skin for a longer period, compared with the commercial patches. Moreover, the patches were able to stick to the skin for more than 24 h in 11 subjects. On the other hand, the commercial product could adhere to the skin for more than 24 h in 2 subjects. These findings are in accordance with the in vitro skin adhesion test (probe tack test). Therefore, the ketoprofen-loaded PVPAA/PMVEMA patches demonstrated an excellent skin adhesion performance, and the patches may be appropriate for transdermal drug delivery.

## 4. Conclusions

Water-based or hydrogel PSAs are naturally compatible with many substances and can moisturize the skin, thus leading to an effective transdermal patch, without the use of a permeation enhancer. PVPAA was successfully synthesized using a chain polymerization reaction between AA and NVP. PVPAA/PMVEMA PSAs were prepared by crosslinking with TA and DAAA, without the use of an organic solvent. The PVPAA/PMVEMA patches prepared at a PVPAA: PMVEMA weight ratio of 1:1 demonstrated the highest adhesive capability due to the optimal ratio of the polymers for metal-coordination complex formation. With the use of both polymers, an easy and painless removal of the patches was achieved. Moreover, the ketoprofen-loaded PVPAA/PMVEMA patches exhibited a comparable permeability coefficient and permeated flux and a superior skin adhesion performance, compared with the commercial patches. Furthermore, the patches did not cause skin irritation in healthy volunteers. Therefore, the PVPAA/PMVEMA PSAs are a promising material for delivering ketoprofen through the skin.

## Figures and Tables

**Table 1:** Synthesis yields of PVPAA.

Polymer	V50 (%wt)	% Yields
PVPAA-01	0.05	21.42 ± 0.25
PVPAA-02	0.1	4.57 ± 0.14
PVPAA-03	0.2	0.55 ± 0.89

**Table 2:** Adhesive properties of the PVPAA/PMVEMA patches (F1–F3), the ketoprofen-loaded patches, and the commercial patch. * Statistically significant difference compared with F2 (*p* < 0.05), ** Statistically significant difference compared with the commercial ketoprofen patches (*p* < 0.05).

Formulation	Ratio of PVPAA: PMVEMA	Tacking Strength (N)	Peeling Strength (N)
F1	0:1	0.12 * ± 0.00	0.07 ± 0.01
F2	1:1	0.33 ± 0.02	0.07 ± 0.01
F3	1:2	0.22 * ± 0.01	0.07 ± 0.01
Ketoprofen-loaded PVPAA/PMVEMA patches	1:1	0.24 ** ± 0.00	0.09 ± 0.01
Commercial (Hisamitsu^®^)	-	0.08 ± 0.01	0.06 ± 0.01

**Table 3:** Skin permeation parameters and drug content of the drug-loaded patches and the commercial patches.

Formulation	Drug Content (%)	Steady-State Flux (µg/cm^2^/h)	Permeability Coefficient (cm/h)
Ketoprofen-loaded patches	98.98 ± 0.58	4.77 ± 1.00	8.15 ± 1.00
Commercial (Hisamitsu^®^)	98.78 ± 0.97	4.33 ± 0.80	8.99 ± 0.80

**Table 4:** Stability study of the ketoprofen-loaded PVPAA/PMVEMA patches.

Time (Months)	Study	Parameters			
		Physical Appearance	Tacking Strength (N)	Peeling Strength (N)	Drug Content (%)
0		colorless hydrogels	0.445 ± 0.03	0.136 ± 0.01	95.39 ± 0.73
3	accelerated	colorless hydrogels	0.409 ± 0.06	0.137 ± 0.01	94.85 ± 0.83
	long-term	colorless hydrogels	0.454 ± 0.01	0.134 ± 0.00	94.55 ± 0.51
6	accelerated	colorless hydrogels	0.494 ± 0.08	0.125 ± 0.00	94.25 ± 0.58
	long-term	colorless hydrogels	0.503 ± 0.04	0.128 ± 0.01	94.36 ± 0.74

## Data Availability

Not applicable.
